# Longitudinal Interplay Between Team Resilience and Team Stress in Low‐Stability Clinical Nursing Teams: A Cross‐Lagged Panel Network Analysis

**DOI:** 10.1155/jonm/7071620

**Published:** 2026-06-19

**Authors:** Zhiwei Wang, Lan Gao, Siya Meng, Xueqing Song, Xiaorong Luan

**Affiliations:** ^1^ School of Nursing and Rehabilitation, Shandong University, Qilu Hospital of Shandong University, Jinan, Shandong, China, qiluhospital.com; ^2^ University of Health and Rehabilitation Sciences, Qingdao, Shandong, China

**Keywords:** cross-lagged panel network, digital health technology burden, moral distress, nurses, team resilience, team stress

## Abstract

**Aim:**

To estimate longitudinal predictive relationships between team resilience and team stress among low‐stability clinical nursing teams, identifying core driving factors and bridging mechanisms using cross‐lagged panel network analysis.

**Background:**

Chronic instability in clinical nursing teams disrupts workflows and triggers systemic team stress, which is further exacerbated by digital health technology burdens and moral distress. Understanding how specific dimensions of team resilience interact with these facets of team stress over time is essential for developing precise organizational interventions.

**Design:**

A two‐wave longitudinal panel study.

**Methods:**

Data from the Nurse Team Health Management Research Cohort across two waves (October 2024 and December 2025) included 5164 clinical nurses aggregated into 285 low‐stability nurse teams. Team resilience and team stress were assessed using the Analyzing and Developing Adaptability and Performance in Teams to Enhance Resilience Scale and a customized Nursing Job Stressor Inventory. A cross‐lagged panel network was estimated to identify influential nodes and network conduits.

**Results:**

Digital health technology burden exhibited the highest predictive power, driving team stress and significantly predicting subsequent moral distress. Moral distress emerged as a potentially destructive bridge, which may negatively predict multiple resilience dimensions over time. Conversely, cooperation with other departments appeared to serve as a protective bridge, mitigating subsequent subjective work stress and digital technology burden. Monitoring exhibited a potentially paradoxical effect, positively predicting subsequent subjective work stress.

**Conclusion:**

Digital health technology burden drives occupational stress in low‐stability nursing teams. Moral distress may erode team resilience, whereas cross‐departmental cooperation acts as a potential protective shield.

**Implications for Nursing Management:**

This study shifts the focus of occupational stress management from the individual to the team level. To stabilize nursing teams facing chronic instability and high turnover, healthcare administrators should move beyond generic stress reduction. Targeted interventions must focus on alleviating digital workflow burdens, instituting routine ethical debriefings to resolve moral distress, and formalizing boundary‐spanning cooperation protocols.

## 1. Introduction

The contemporary healthcare environment is characterized by unprecedented volatility, largely driven by severe nursing shortages and high turnover rates [1]. Consequently, clinical nursing teams frequently operate in a state of “low stability,” defined by the continuous departure, transfer, or retirement of core team members [2, 3]. Because these clinical units are intended to be enduring, intact organizational structures, chronic turnover forces a continuous state of flux that repeatedly fractures established workflows, shared mental models, and interpersonal trust [3]. This disruption diminishes collective efficacy and places an immense adaptive burden on the remaining staff [2, 3]. Within this low‐stability context, occupational stress is no longer merely an individual psychological phenomenon; it manifests as a systemic “team stress” that threatens the overall safety and quality of patient care [3, 4].

Modern clinical settings have introduced novel dimensions to this collective stress. Beyond traditional subjective work stress, nurses are increasingly burdened by digital health technology [5]. While electronic health records (EHRs) and digital tools were designed to enhance efficiency, they often dominate direct patient care time, leading to severe operational burnout [5, 6]. Furthermore, chronic understaffing in low‐stability teams frequently triggers moral distress—a collective sense of guilt and frustration when institutional constraints prevent the team from delivering ethically and clinically optimal care [7–9]. Addressing these multidimensional team stressors is a critical priority for nursing management.

## 2. Background

To navigate and withstand such profound systemic stressors, individual psychological hardiness is insufficient; “team resilience” is paramount [10, 11]. Team resilience is conceptualized as a dynamic, collective capability that enables a group to positively adapt to adversity, maintain team functioning, and recover from setbacks [3, 10–12]. Unlike static traits, it involves complex, interactive processes [13]. According to the analyzing and developing adaptability and performance in teams to enhance resilience (ADAPTER) framework, team resilience encompasses multiple behavioral and cognitive dimensions, including responding, shared transformational leadership, learning, anticipating, monitoring, cooperation with other departments, and heedful interrelating [14]. Understanding how these specific, granular dimensions of team resilience interact with distinct facets of team stress is essential for developing precise organizational interventions [10].

Despite the recognized importance of resilience in mitigating occupational stress, several critical methodological and conceptual gaps persist in the existing literature. First, the vast majority of studies have focused on the individual level, overlooking the reality that nurses operate within highly interdependent teams [15, 16]. Second, traditional latent variable models (e.g., structural equation modeling) treat resilience and stress as monolithic constructs [17, 18]. This approach obscures granular, dimension‐to‐dimension interactions, making it impossible to determine, for instance, whether cross‐departmental cooperation explicitly buffers digital technology burden [19]. Third, most existing studies rely on cross‐sectional data, making it impossible to establish the directional, temporal mechanisms through which these dimensions influence one another over time [19, 20].

To overcome these limitations, the network analysis approach has emerged as a paradigm‐shifting methodology in psychology and organizational behavior [19, 21]. The network perspective conceptualizes psychological and behavioral phenomena not as latent constructs but as complex systems of mutually interacting symptoms or dimensions (nodes) connected by predictive pathways (edges) [19, 21]. Recently, cross‐lagged panel network (CLPN) analysis has advanced this framework by incorporating longitudinal data, allowing researchers to disentangle the bidirectional, temporal dynamics between different symptom communities over time [22–24]. By calculating metrics such as out‐expected influence (Out‐EI), CLPN can pinpoint the “core driver” nodes that initiate cascades within the network [22–24]. Furthermore, evaluating “bridge expected influence” (BEI) allows for the identification of specific symptoms that act as active transmitters or protective shields connecting two distinct communities (e.g., the team stress community and the team resilience community) [20]. Given the chronic instability of clinical nursing teams, applying CLPN to team‐level aggregated data provides an unprecedented opportunity to map the structural and processual mechanisms underlying the interplay between team resilience and stress.

Addressing the critical gaps identified above, the current study aimed to explore the dynamic, longitudinal interplay between team resilience and team stress among low‐stability clinical nursing teams. Specifically, this study pursued three primary objectives: (1) to visualize the temporal, dimension‐to‐dimension network structure between team resilience and team stress over time; (2) to identify the core driving dimensions that exert the greatest predictive impact on the overall network evolution; and (3) to pinpoint the critical bridge nodes. By achieving these aims, this study seeks to provide targeted, high‐yield intervention strategies for nursing administrators to stabilize and empower teams facing chronic instability.

## 3. Methods

### 3.1. Study Design and Participants

Employing a two‐wave longitudinal panel design, data for this longitudinal study were drawn from the “Nurse Team Health Management Research Cohort,” the first established cohort of its kind in China. This ongoing cohort covers hospitals across various healthcare service tiers in all 16 prefecture‐level cities of Shandong Province. Since 2020, annual surveys have been conducted using a stratified convenience sampling approach. To investigate the dynamic relationships between team resilience and team stress, the current study utilized datasets from the October 2024 (Time point 1) and December 2025 (Time point 2) waves.

To ensure sample homogeneity and clinical relevance, a two‐level inclusion strategy was implemented. At the team level, to accurately capture the targeted “low‐stability” context, the study exclusively included teams that had experienced the departure, transfer, or retirement of core members (defined as official members with more than 1 year of postprobation tenure within the team) within the past year. At the individual level, participants were enrolled based on the following inclusion criteria: (1) holding a valid “Nurse Practice Certificate of the People’s Republic of China”; and (2) having successfully completed their clinical probation period. Nurses who were not directly engaged in bedside patient care (e.g., those in purely administrative, logistical, or educational roles) were strictly excluded from the analysis. All participants provided informed consent prior to data collection.

The final analytical sample consisted of 5164 clinical nurses nested within 285 teams; no missing values were observed for any focal variables. Operationally, a “nurse team” was defined as a group of nurses collaborating within the same clinical department or ward, sharing joint responsibility for comprehensive patient care. Following methodological guidance for multilevel research, a robust team‐level study requires a minimum of 51 teams, with at least 5 individual members per team to ensure adequate statistical power [25]. Every included team successfully met this minimum threshold, with team sizes ranging specifically from 5 to 190 members, thereby fully satisfying these methodological requirements. The study was reported in accordance with the Strengthening the Reporting of Observational Studies in Epidemiology (STROBE) guidelines.

### 3.2. Measures

#### 3.2.1. Team Resilience

Team resilience was assessed using the ADAPTER scale [4, 14]. The scale comprises 51 items distributed across seven dimensions: responding, shared transformational leadership, learning, anticipating, monitoring, cooperation with other departments, and heedful interrelating. Responses were recorded on a 5‐point Likert scale, ranging from 1 (*strongly disagree*) to 5 (*strongly agree*). To facilitate network analysis and ensure comparability across nodes, the total score for each dimension was divided by the number of its constituent items, yielding a mean item score for each dimension. In the current sample, Cronbach’s *α* coefficients for the seven dimensions ranged from 0.766 to 0.984.

#### 3.2.2. Team Stress

Team stress was evaluated using a customized 3‐item tool derived from the Nursing Job Stressor Inventory (NJSI) [26]. Each item represented a distinct dimension of team stress: (1) Subjective work stress: Measured via a single‐item numeric rating scale assessing overall daily work stress, ranging from 0 (*no stress*) to 10 (*extreme stress*). (2) Moral distress: Assessed the frequency of feeling guilty or helpless due to the inability to provide ideal patient care caused by resource limitations or external pressures (e.g., hospital policies) over the past month. Responses were scored on a 5‐point scale (1 = *never* to 5 = *very frequently*). (3) Digital health technology burden: Evaluated whether the use of digital tools (e.g., electronic medical records) significantly occupied direct nursing time, leading to operational burnout or reduced patient communication quality. Responses were rated on a 5‐point Likert scale (1 = *completely disagree* to 5 = *completely agree*). Notably, these three specific dimensions were prioritized because they represent the most salient, systemic environmental constraints affecting modern clinical workflows [5–9]. Rather than capturing idiosyncratic personal anxieties, these items reflect shared structural burdens—such as pervasive unit understaffing, collective ethical challenges, and mandatory hospital‐wide digital protocols. Consequently, they accurately align with the conceptualization of occupational stress as a collective “team stress” phenomenon.

### 3.3. Data Aggregation

Given that team resilience and team stress in this study are conceptualized as shared perceptions and collective states among team members, individual‐level responses from the 5164 nurses were aggregated to the team level (i.e., calculating the mean score of members within each of the 285 teams) [19, 27]. To statistically justify the appropriateness of this aggregation, inter‐rater agreement (*r*_wg_) and intraclass correlation coefficients (ICC (1) and ICC (2)) were evaluated. Conventional methodological standards suggest that *r*_wg_ > 0.70, ICC (1) > 0.05, and ICC (2) > 0.50 indicate acceptable within‐group agreement and between‐group variance for aggregation [28, 29]. Across both time points in this study, the *r*_wg_ values for all dimensions of team resilience and team stress consistently exceeded 0.9. Concurrently, the ICC (1) values were all greater than 0.1, and the ICC (2) values all exceeded 0.6. These robust metrics provided strong empirical support for aggregating the individual nurse data into 285 team‐level cases for subsequent network analysis.

### 3.4. Statistical Analysis

Descriptive statistics, reliability analyses, and data aggregation evaluations were conducted using SPSS Version 25.0 and the SPSSAU online platform. All CLPN analyses were performed using R software (Version 4.4.1).

#### 3.4.1. CLPN Estimation

To explore the longitudinal interactions between team resilience and team stress, a CLPN was estimated using the glmnet package [30]. To isolate the specific temporal dynamics between our core variables, team size was incorporated into the model as a covariate (specifically, as an additional predictor), thereby controlling for its potential confounding influence on team‐level interactions. The network edges were regularized via the least absolute shrinkage and selection operator (LASSO) to shrink small, spurious partial correlations to exactly zero, thereby enhancing model interpretability and preventing overfitting [31]. The network was visualized using the qgraph package, where nodes represent symptoms and edges represent predictive relationships over time [32, 33].

#### 3.4.2. Centrality and Bridge Centrality Estimation

To identify the most influential nodes driving the network, Out‐EI and in‐expected influence (In‐EI) were calculated [34]. Out‐EI quantifies the extent to which a specific node at Time 1 predicts other nodes at Time 2, whereas In‐EI measures a node’s susceptibility to being predicted by other network components. Furthermore, to elucidate the mechanisms of interaction between the two distinct communities (team resilience and team stress), BEI (1‐step) was computed using the networktools package [35]. A high BEI indicates that a node plays a pivotal role in transferring effects across community boundaries [36].

#### 3.4.3. Network Stability and Accuracy

The robustness of the network was rigorously tested using the bootnet package [37]. First, the accuracy of edge weight estimates was assessed by generating 95% confidence intervals via nonparametric bootstrapping (1000 samples). Second, the stability of the centrality indices (Out‐EI, In‐EI, and BEI) was evaluated using a case‐dropping bootstrap procedure. The correlation stability (CS) coefficient was calculated, representing the maximum proportion of cases that can be dropped to retain a correlation of at least 0.7 with the original centrality metrics (a CS coefficient > 0.25 is generally considered acceptable) [38]. Finally, bootstrapped difference tests (*α* = 0.05) were conducted to determine whether edge weights and node centralities differed significantly from one another.

### 3.5. Ethical Considerations

All study procedures were conducted in strict adherence to the ethical principles outlined in the Declaration of Helsinki. The research protocol was thoroughly reviewed and formally approved by the Ethics Committee of Scientific Research of Shandong University School of Nursing and Rehabilitation (Ethics Approval No. 2021‐R‐131). Participation was entirely voluntary, and as previously stated, informed consent was obtained from all participants prior to data collection. Furthermore, strict confidentiality and anonymity protocols were maintained throughout the study, ensuring that all individual and team‐level responses were securely stored and used solely for research purposes.

## 4. Results

### 4.1. Sample Characteristics

A total of 5164 clinical nurses nested within 285 teams were included in this study. The mean age of the participants was 34.52 ± 6.72 years, with an average of 12.19 ± 7.46 years of working experience. The majority of the sample was female (88.8%) and married (80.8%). Regarding educational attainment, 94.1% held a bachelor’s degree, while 1.1% held a master’s degree or above. Professionally, exactly half of the participants (50.2%) held the title of nurse‐in‐charge, followed by senior nurses (32.8%). Further details regarding the sociodemographic characteristics of the participants are presented in Supporting Table S1.

### 4.2. CLPN Structure

**FIGURE 1:**
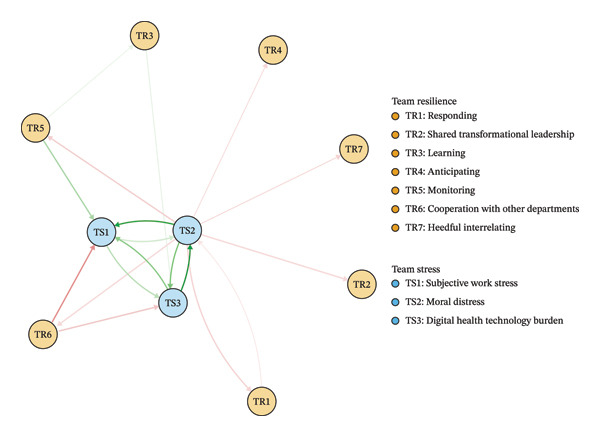
The cross‐lagged panel network of team resilience and team stress among nurses. Note. Nodes represent the seven dimensions of team resilience (TR1–TR7) and the three dimensions of team stress (TS1–TS3). The arrows indicate cross‐lagged predictive effects from Time 1 to Time 2. Green edges indicate positive relationships, whereas red edges indicate negative relationships. The thickness of the edges reflects the strength of the predictive weights. To enhance visual interpretability, autoregressive edges and cross‐lagged paths with absolute weights ≤ 0.04 have been omitted from this visualization. The three most influential cross‐community pathways are as follows: cooperation with other departments (TR6) negatively predicting subjective work stress (TS1; weight = −0.227); monitoring (TR5) positively predicting subjective work stress (TS1; weight = 0.174); and cooperation with other departments (TR6) negatively predicting digital health technology burden (TS3; weight = −0.115).

The CLPN model estimated the longitudinal relationships between the seven dimensions of team resilience (TR1–TR7) and the three dimensions of team stress (TS1–TS3) across two time points (see Supporting Table S2 for the full adjacency matrix). Autoregressive coefficients (represented by bold values on the diagonal of the adjacency matrix and self‐loops in the full network, as shown in Supporting Figure S1) indicated moderate to strong temporal stability for variables such as digital health technology burden (TS3 to TS3: 0.540) and subjective work stress (TS1 to TS1: 0.302). To enhance the visual interpretability of the most relevant cross‐lagged effects, autoregressive edges and cross‐lagged paths with absolute weights ≤ 0.04 were removed from the main network visualization (Figure 1).

To elucidate the dynamic interplay between communities, cross‐lagged effects (off‐diagonal values) were examined. A notable bidirectional mechanism emerged between team stress and team resilience. As illustrated in Figure 1, moral distress at Time 1 (TS2) served as a significant negative predictor for multiple team resilience dimensions at Time 2, including monitoring (TR5: −0.096), heedful interrelating (TR7: −0.094), responding (TR1: −0.086), anticipating (TR4: −0.083), shared transformational leadership (TR2: −0.074), and cooperation with other departments (TR6: −0.074). Conversely, cooperation with other departments at Time 1 (TR6) negatively predicted subsequent subjective work stress (TS1: −0.227) and digital health technology burden (TS3: −0.115).

Additionally, strong positive predictive pathways were observed within the team stress community itself, where digital health technology burden at Time 1 (TS3) strongly predicted moral distress at Time 2 (TS2: 0.514). Notably, monitoring at Time 1 (TR5) exhibited a positive cross‐lagged effect on subjective work stress at Time 2 (TS1: 0.174).

### 4.3. Centrality and Bridge Centrality Estimates

**FIGURE 2:**
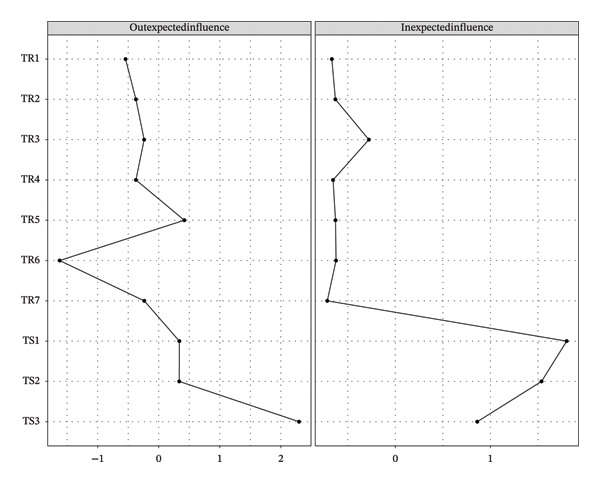
Centrality estimates of the cross‐lagged panel network. Note. The figure illustrates the out‐expected influence (left panel) and in‐expected influence (right panel) for each node within the network. Values are presented as standardized z‐scores on the *x*‐axis. Out‐expected influence reflects the degree to which a specific variable at Time 1 predicts other variables at Time 2, whereas in‐expected influence indicates the extent to which a variable at Time 2 is predicted by others. TR1–TR7 represent the seven dimensions of team resilience; TS1–TS3 represent the three dimensions of team stress.

To identify the most influential nodes driving the network, Out‐EI and In‐EI were calculated (see Table 1 and Figure 2). Digital health technology burden (TS3) exhibited the highest predictive power across the network (Out‐EI = 2.297), followed by monitoring (TR5, Out‐EI = 0.419). Conversely, cooperation with other departments (TR6) exhibited the strongest negative predictive influence across the network (Out‐EI = −1.623), indicating its crucial role in mitigating subsequent team stress. Regarding susceptibility to the influence of other variables, as illustrated in Figure 2, subjective work stress (TS1) recorded the highest In‐EI (1.802), closely followed by moral distress (TS2, In‐EI = 1.537).

**TABLE 1:** Descriptive statistics of the nodes in the cross‐lagged panel network.

Nodes	Mean (SD)		Out‐EI	In‐EI	BEI
	Time point 1	Time point 2			
TR1: Responding	4.648 (0.236)	4.738 (0.201)	−0.544	−0.668	0.128
TR2: Shared transformational leadership	4.641 (0.238)	4.716 (0.201)	−0.374	−0.631	0.360
TR3: Learning	4.570 (0.281)	4.549 (0.239)	−0.239	−0.279	0.544
TR4: Anticipating	4.657 (0.228)	4.722 (0.200)	−0.374	−0.655	0.360
TR5: Monitoring	4.636 (0.241)	4.707 (0.210)	0.419	−0.629	1.202
TR6: Cooperation with other departments	4.655 (0.225)	4.702 (0.199)	−1.623	−0.624	−1.344
TR7: Heedful interrelating	4.648 (0.243)	4.722 (0.206)	−0.237	−0.716	0.360
TS1: Subjective work stress	7.434 (0.532)	6.886 (0.642)	0.337	1.802	0.235
TS2: Moral distress	2.904 (0.618)	2.363 (0.609)	0.336	1.537	−2.205
TS3: Digital health technology burden	2.639 (0.605)	3.265 (0.630)	2.297	0.861	0.360

*Note:* TR1–TR7 represent the seven dimensions of team resilience; TS1–TS3 represent the three dimensions of team stress.

Abbreviations: BEI: bridge expected influence; EI: expected influence; SD: standard deviation; Out‐EI, In‐EI, and BEI values are presented as standardized z‐scores.

**FIGURE 3:**
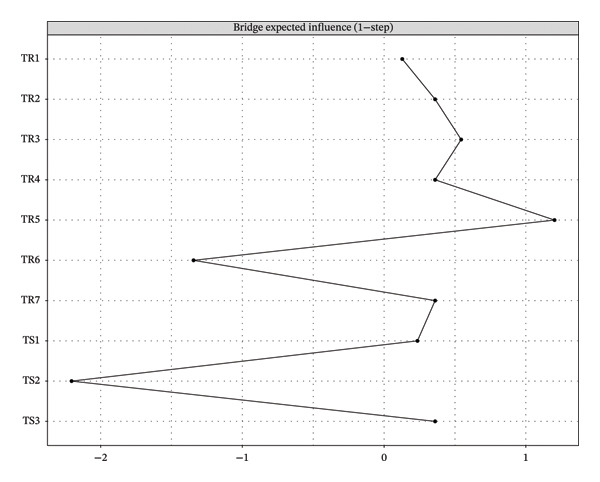
Standardized bridge expected influence of the nodes in the cross‐lagged panel network. Note. The figure displays the bridge expected influence (1‐step) for each node, expressed as standardized z‐scores on the *x*‐axis. Bridge expected influence quantifies the cumulative strength of a node’s edges connecting exclusively to nodes in the opposing community, thereby highlighting its role in transferring effects between team resilience and team stress. TR1–TR7 represent the seven dimensions of team resilience; TS1–TS3 represent the three dimensions of team stress.

BEI was assessed to determine which symptoms act as primary conduits connecting the team resilience and team stress communities (Figure 3). Based on the standardized indices, monitoring (TR5) emerged as the strongest positive bridge node (BEI = 1.202), whereas moral distress (TS2, BEI = −2.205) and cooperation with other departments (TR6, BEI = −1.344) exhibited the strongest negative bridge influences. To further disentangle the directional nature of these cross‐community interactions, bridge Out‐EI and In‐EI were evaluated (Supporting Figure S2), alongside a focused visualization of exclusively cross‐community pathways (Supporting Figure S3). The decomposition of bridge centrality revealed that the high overall bridge capacity of TR5, TS2, and TR6 was primarily characterized by their driving effects on the opposing community (i.e., pronounced bridge Out‐EI). This suggests that these specific dimensions may act primarily as active transmitters rather than passive receivers, highlighting their potential mechanistic roles in propagating or mitigating effects between team stress and team resilience.

### 4.4. Network Stability and Accuracy

The robustness of the network model was rigorously evaluated. Nonparametric bootstrapping (1000 samples) confirmed the accuracy of the edge weight estimates (Supporting Figure S4), demonstrating that the 95% confidence intervals were sufficiently narrow and consistent around the sample mean. A bootstrapped difference test (*α* = 0.05) further verified significant differences among individual edge weights (Supporting Figure S5).

The case‐dropping bootstrap procedure was employed to assess the stability of the node influence indices (Supporting Figure S6). The CS coefficients exceeded the recommended minimum threshold of 0.25 for In‐EI (CS = 0.751) and Out‐EI (CS = 0.361), indicating acceptable to high reliability even after dropping portions of the sample data. However, the CS coefficient for BEI was 0.207, which falls slightly below the ideal 0.25 cutoff. Consequently, while the core directional centralities are robust, the specific bridge centrality rankings should be interpreted with some caution. Additionally, bootstrapped difference tests for the node influence indices confirmed that the high centrality values of core nodes (e.g., TS3 and TR5) were statistically distinct from those of the less central nodes (Supporting Figures S7‐S9).

## 5. Discussion

To our knowledge, this is the first study to explore the dynamic, longitudinal interplay between team resilience and team stress among clinical nurses in low‐stability teams using a CLPN approach. By relying on a large‐scale, team‐level aggregated cohort, this study moves beyond individual‐level psychological assessments to identify the core drivers and cross‐community bridges that shape team‐level occupational health over time.

### 5.1. Digital Health Technology Burden (TS3): The Core Driver of Team Stress

Our network analysis revealed that digital health technology burden (TS3) exhibited the highest Out‐EI (Out‐EI = 2.297) across the network. Longitudinally, TS3 at Time 1 emerged as a powerful positive predictor of moral distress (TS2) at Time 2 (edge weight = 0.514). In the context of this study, digital health technology burden (TS3) refers to the excessive cognitive and temporal demands placed on nurses by the mandatory use of EHRs and other digital tools [39, 40]. It represents a systemic stressor where technology, originally designed to assist, ends up dominating direct patient care time and causing operational burnout [39, 40].

This finding aligns with emerging evidence suggesting that technology‐induced stress is becoming a primary occupational hazard in modern healthcare [39–42]. Previous studies have indicated that poorly integrated digital systems force nurses to focus on screens rather than patients, disrupting the nurse–patient therapeutic relationship [41, 42]. In the specific context of low‐stability teams, this burden is exponentially magnified. When core members leave, remaining staff face increased patient loads and spend excessive time training temporary staff on complex digital workflows [43]. Consequently, the inability to balance mandatory data entry with holistic patient care fuels subsequent moral distress (TS2). Nurses feel they are compromising ethical standards due to systemic technological constraints.

### 5.2. Moral Distress (TS2): The Destructive Bridge Eroding Team Resilience

Moral distress (TS2) demonstrated the strongest negative BEI (BEI = −2.205). It emerged as a probable active transmitter, with findings suggesting that it may negatively predict nearly all dimensions of team resilience at Time 2, including monitoring (TR5), responding (TR1), and shared transformational leadership (TR2). Moral distress (TS2) captures the collective guilt and helplessness experienced when institutional constraints (e.g., severe understaffing) prevent a team from delivering clinically and ethically required care [7–9]. While previous literature often treats moral distress as an individual psychological outcome, our network highlights its role as a destructive, team‐level “corrosive agent.” Research has shown that unresolved moral distress leads to emotional withdrawal, decreased communication, and higher turnover intentions [7–9]. In low‐stability teams, where trust and routines are already fragile due to staff turnover, chronic moral distress has the potential to severely impair the team’s adaptive capacities. When collective ethical efforts feel futile, motivation to engage in proactive resilience behaviors—such as mutual monitoring (TR5) or shared leadership (TR2)—is profoundly suppressed over time.

### 5.3. Cooperation With Other Departments (TR6): The Protective Shield

Cooperation with other departments (TR6) exhibited a significant negative BEI (BEI = −1.344), potentially acting as a primary mitigating factor associated with a probable reduction in subsequent subjective work stress (TS1) and digital health technology burden (TS3). TR6 represents the team’s boundary‐spanning capacity—their ability to effectively communicate, share resources, and coordinate care workflows with external departments (e.g., pharmacy, logistics, or specialized units) rather than operating in isolation [14]. This finding extends the traditional internal focus of team resilience by emphasizing external socio‐technical networks. Existing organizational behavior studies suggest that boundary‐spanning activities can buffer internal resource depletion [44]. For low‐stability teams struggling with internal staffing deficits, effective cross‐departmental cooperation could serve as a crucial compensatory mechanism [45, 46]. By streamlining patient transfers and sharing workloads with other departments, teams may significantly reduce the chaotic bottlenecks that exacerbate subjective stress and digital documentation burdens.

### 5.4. The Paradoxical Effect of Monitoring (TR5): From Vigilance to Vulnerability

Although monitoring (TR5) was the strongest positive bridge node (BEI = 1.202), its predictive effect was paradoxical: TR5 at Time 1 positively predicted subjective work stress (TS1) at Time 2 (edge weight = 0.174). Monitoring (TR5) involves team members continuously observing each other’s actions, maintaining situational awareness, and catching potential clinical errors before they reach the patient [14, 19]. It is traditionally viewed as a core component of high‐reliability teams [14]. The positive cross‐lagged pathway from monitoring to stress suggests a probable “dark side” of resilience behaviors in vulnerable teams. While mutual monitoring is essential for patient safety, previous research indicates that excessive vigilance in a low‐trust or under‐resourced environment can be perceived as micromanagement [47, 48]. In low‐stability teams, integrating unfamiliar members necessitates hypervigilance to prevent adverse events; however, this prolonged alertness is cognitively exhausting. Over time, intense monitoring can create a psychologically unsafe environment, leaving nurses feeling scrutinized and driving up overall subjective work stress [49].

### 5.5. Clinical Implications and Interventions

Based on the identified core drivers and potential bridging mechanisms, nursing administrators should shift from generic stress‐reduction programs to a prioritized hierarchy of targeted, node‐specific interventions to stabilize low‐stability teams.

#### 5.5.1. Targeting the Core Stress Driver: Digital Health Technology Burden (TS3)

Short‐term operational actions: Administrators should conduct immediate “digital workflow audits.” During shift changes or peak admission times, executive leaders should deploy dedicated clinical informatics nurses (super‐users) to directly assist temporary or new staff with EHR documentation, thereby immediately alleviating frontline cognitive load.

Long‐term structural investments: Hospitals must invest in optimizing EHR user interfaces to reduce repetitive data entry and streamline documentation, aiming for a measurable reduction in daily EHR documentation time (e.g., a 15%–20% decrease per shift) [50]. This requires sustained collaboration between nursing leadership and information technology departments to ensure technology serves as an enabler rather than an erosive distractor over time [50].

#### 5.5.2. Mitigating the Erosive Mechanism: Moral Distress (TS2)

Short‐term operational actions: To help mitigate the potential for moral distress to erode team resilience, hospitals should establish routine “Schwartz Center Rounds” or similar ethics‐focused debriefing sessions [51, 52]. Particularly after periods of severe understaffing or high patient mortality, providing a psychologically safe space for the team to collectively process ethical conflicts can halt the immediate transition from distress to team‐level withdrawal.

Long‐term structural investments: Executive leaders should develop proactive, unit‐level ethical resource frameworks. This involves structurally empowering clinical ethics committees to be accessible on the ward level, ensuring that frontline nurses have sustained, formalized support systems when facing chronic resource limitations.

#### 5.5.3. Fortifying Protective Buffers: Cross‐Departmental Cooperation (TR6) and Psychological Safety (TR5)

Short‐term operational actions: To prevent the paradoxical stress potentially induced by hypervigilance (TR5), immediate team training (such as TeamSTEPPS) should be implemented to reframe mutual monitoring [53]. Leaders must actively cultivate a “just culture” where cross‐checking is explicitly framed as mutual support and error‐trapping, rather than punitive surveillance. The success of this cultural shift can be quantitatively tracked through improvements in routine team‐level psychological safety scores or a documented reduction in unreported near‐miss events. Simultaneously, daily interdisciplinary “huddles” should be initiated to immediately bridge communication gaps between wards (TR6).

Long‐term structural investments: Hospital leadership must formalize and fund boundary‐spanning protocols (TR6). This includes creating permanent liaison nurse positions to manage cross‐departmental workflows (e.g., coordinating with pharmacy and logistics). Institutionalizing these external support systems may act as a crucial, sustainable buffer against the internal chaos experienced by low‐stability teams.

### 5.6. Limitations

Several limitations must be acknowledged when interpreting these findings. First, the network structures and specific pathways identified are inherently dependent on the specific instruments utilized in this study (i.e., the ADAPTER scale and the customized NJSI items). While these tools are highly validated for our context, employing alternative instruments with different dimensional conceptualizations of resilience or stress might yield different network topologies. Specifically, the assessment of team stress relied on three brief, customized indicators. Although this pragmatic strategy was prioritized to mitigate survey fatigue within high‐workload clinical settings and was supported by robust aggregation metrics, the use of brief or single‐item measures inherently constrains the depth and nuance with which complex stress dimensions are captured compared to comprehensive psychometric scales. Future research should therefore replicate these models using diverse, multidimensional assessment tools to further validate the findings. Second, the CS coefficient for BEI (CS = 0.207) fell slightly below the ideal methodological threshold (0.25). Although we strictly adhered to case‐dropping bootstrapping to report this transparently and ensure methodological rigor, the specific rankings of bridge nodes should be interpreted with caution. Therefore, our interpretations regarding the bridging dynamics should be framed as probable mechanisms rather than definitive pathways, and these specific structural relationships warrant rigorous replication in future independent cohorts. Future studies should aim to secure even larger team‐level sample sizes to enhance the stability of cross‐community metrics. Third, while our data were rigorously aggregated to the team level using inter‐rater agreement (r_wg_) and ICC metrics to mitigate individual subjectivity, the reliance on self‐reported questionnaires remains susceptible to common method bias. We made significant efforts to ensure anonymity to encourage honest reporting; nevertheless, future studies should incorporate objective, multisource organizational data (e.g., actual turnover rates, EHR log‐in durations, or adverse event registries) to triangulate and validate the self‐reported network dynamics. Fourth, a significant limitation of this study lies in the crude operationalization of “low‐stability” (or personnel turnover). Due to restricted access to detailed administrative human resource databases, personnel changes were assessed as a binary inclusion criterion (presence vs. absence of core member turnover) rather than a continuous turnover rate. Consequently, this study treats “teams experiencing turnover” as a homogenous group, failing to capture the potential dose–response relationship between the severity of instability (e.g., a 5% vs. 40% turnover rate) and network dynamics. Losing a highly experienced charge nurse undoubtedly disrupts team resilience networks more profoundly than the departure of a junior staff member. Future longitudinal studies must incorporate precise objective turnover metrics—including the volume, frequency, and composition of staff departures—to elucidate how different magnitudes of instability uniquely alter the interplay between team stress and resilience. Fifth, the cross‐cultural generalizability of these findings warrants careful consideration. This study was conducted exclusively within the Chinese healthcare system, which possesses unique organizational and sociocultural characteristics. Specifically, Chinese nursing staffing models often involve higher patient‐to‐nurse ratios and a distinct hierarchical management structure. These context‐specific factors may uniquely amplify the baseline intensity of moral distress and culturally influence the dynamics of mutual monitoring (TR5). Additionally, the specific manifestation of the digital health technology burden (TS3) may be tied to China’s rapid, centralized deployment of specific EHR interfaces. Consequently, the exact magnitude and edge weights of these specific network pathways may be context‐dependent. However, we posit that the fundamental structural mechanisms identified—namely, that excessive digital workflow burdens drive systemic team stress and that moral distress acts as a destructive bridge eroding collective resilience—are highly transferable to other international nursing environments. These core pathways align closely with universal workforce challenges, such as widespread EHR‐related burnout and global nursing shortages. Future cross‐cultural comparative studies are highly recommended to delineate which network dynamics are culturally universal and which are uniquely shaped by local healthcare systems.

## 6. Conclusions

In conclusion, this CLPN analysis provides a novel, dynamic perspective on how low‐stability nursing teams function. Digital technology burden acts as the primary catalyst for team stress, while moral distress and cross‐departmental cooperation emerge as probable critical bridges that may respectively erode and protect team resilience. By targeting these specific influential nodes, healthcare administrators can implement precise, high‐yield interventions to support nursing teams navigating the challenges of high staff turnover and clinical instability.

## Author Contributions

Zhiwei Wang: conceptualization, methodology, formal analysis, and writing–original draft. Lan Gao: investigation, data curation, and writing–review and editing. Siya Meng: investigation, data curation, and writing–review and editing. Xueqing Song: investigation, data curation, and writing–review and editing. Xiaorong Luan: conceptualization, supervision, project administration, and writing–review and editing.

## Funding

This work was supported by the Provincial Key Research and Development Program of Shandong [grant number 2021CXGC011301].

## Ethics Statement

All study procedures were conducted in strict adherence to the ethical principles outlined in the Declaration of Helsinki. The research protocol was approved by the Ethics Committee of Scientific Research of Shandong University School of Nursing and Rehabilitation (Ethics Approval No. 2021‐R‐131). All study participants provided informed consent prior to data collection.

## Conflicts of Interest

The authors declare no conflicts of interest.

## Supporting Information

Additional supporting information can be found online in the Supporting Information section.

## Supporting information


**Supporting Information 1** Supporting File 1: Contains Supporting Table S1 detailing the demographic characteristics of the participants, Supporting Table S2 providing the adjacency matrix for the cross‐lagged panel network, and Supporting Figures S1–S9 illustrating the estimated network structures, bridge expected influences, and the accuracy and stability test results of the network estimations.

**Supporting Information 2** Supporting File 2: STROBE statement checklist, which provides the checklist of items included in reports of cohort studies to ensure transparent and standardized reporting. The following supporting information is available for this article.

## Data Availability

The data that support the findings of this study are available from the corresponding author upon reasonable request.
