# Supplementary material for: Longitudinal Interplay Between Team Resilience and Team Stress in Low‐Stability Clinical Nursing Teams: A Cross‐Lagged Panel Network Analysis
Source: J Nurs Manag. 2026 Jun 19;2026:7071620. doi: 10.1155/jonm/7071620 (PMC13282460; doi:10.1155/jonm/7071620)
Supplement: Supplementary file 1 — Supporting Information 1 Supporting File 1: Contains Supporting Table S1 detailing the demographic characteristics of the participants, Supporting Table S2 providing the adjacency matrix for the cross‐lagged panel network, and Supporting Figures S1–S9 illustrating the estimated network structures, bridge expected influences, and the accuracy and stability test results of the network estimations. [file JONM-2026-7071620-s002.docx]

**Table S1** | Demographic characteristics of the participants.

| Variable | Total |
| --- | --- |
|  | (N = 5164) |
| Age (years), Mean ± SD | 34.52 ± 6.72 |
| Years of working experience, Mean ± SD | 12.19 ± 7.46 |
| Gender, n (%) |  |
| Female | 4584 (88.8%) |
| Male | 580 (11.2%) |
| Educational level, n (%) |  |
| Junior college degree or below | 248 (4.8%) |
| Bachelor’s degree | 4857 (94.1%) |
| Master’s degree or above | 59 (1.1%) |
| Marital status, n (%) |  |
| Single | 923 (17.9%) |
| Married | 4175 (80.8%) |
| Divorced or widowed | 66 (1.3%) |
| Number of children, n (%) |  |
| 0 | 1310 (25.4%) |
| 1 | 1603 (31.0%) |
| 2 | 2104 (40.7%) |
| ≥3 | 147 (2.8%) |
| Average monthly income, n (%) |  |
| Very dissatisfied | 148 (2.9%) |
| Dissatisfied | 284 (5.5%) |
| Neutral | 1884 (36.5%) |
| Satisfied | 2096 (40.6%) |
| Very satisfied | 752 (14.6%) |
| Professional title, n (%) |  |
| Nurse | 519 (10.1%) |
| Senior nurse | 1694 (32.8%) |
| Nurse-in-charge | 2594 (50.2%) |
| Associate chief superintendent nurse | 333 (6.4%) |
| Chief superintendent nurse | 24 (0.5%) |

**Table S2** | Adjacency matrix for the cross-lagged panel network.

|  | TR1 | TR2 | TR3 | TR4 | TR5 | TR6 | TR7 | TS1 | TS2 | TS3 |
| --- | --- | --- | --- | --- | --- | --- | --- | --- | --- | --- |
| TR1 | **0.000** | 0.000 | 0.000 | 0.000 | 0.000 | 0.000 | 0.000 | 0.000 | -0.048 | 0.000 |
| TR2 | 0.000 | **0.000** | 0.000 | 0.000 | 0.000 | 0.000 | 0.000 | 0.000 | 0.000 | 0.000 |
| TR3 | 0.000 | 0.000 | **0.000** | 0.000 | 0.000 | 0.000 | 0.000 | -0.026 | 0.000 | 0.064 |
| TR4 | 0.000 | 0.000 | 0.000 | **0.000** | 0.000 | 0.000 | 0.000 | 0.000 | 0.000 | 0.000 |
| TR5 | 0.000 | 0.000 | 0.049 | 0.000 | **0.000** | 0.000 | 0.000 | 0.174 | 0.000 | 0.000 |
| TR6 | 0.000 | 0.000 | 0.000 | 0.000 | 0.000 | **0.000** | 0.000 | -0.227 | -0.010 | -0.115 |
| TR7 | 0.000 | 0.008 | 0.000 | 0.000 | 0.030 | 0.000 | **0.035** | 0.000 | 0.000 | 0.000 |
| TS1 | 0.000 | -0.009 | 0.000 | 0.000 | -0.010 | 0.000 | -0.006 | **0.302** | 0.090 | 0.136 |
| TS2 | -0.086 | -0.074 | -0.024 | -0.083 | -0.096 | -0.074 | -0.094 | 0.462 | **0.302** | 0.268 |
| TS3 | 0.000 | 0.000 | 0.000 | 0.000 | 0.000 | 0.000 | 0.000 | 0.239 | 0.514 | **0.540** |

Note. Rows represent nodes at Time 1, and columns represent nodes at Time 2. Values represent edge weights. Bold values on the diagonal represent autoregressive coefficients; off-diagonal values represent cross-lagged effects. TR1-TR7: seven dimensions of team resilience; TS1-TS3: three dimensions of team stress.


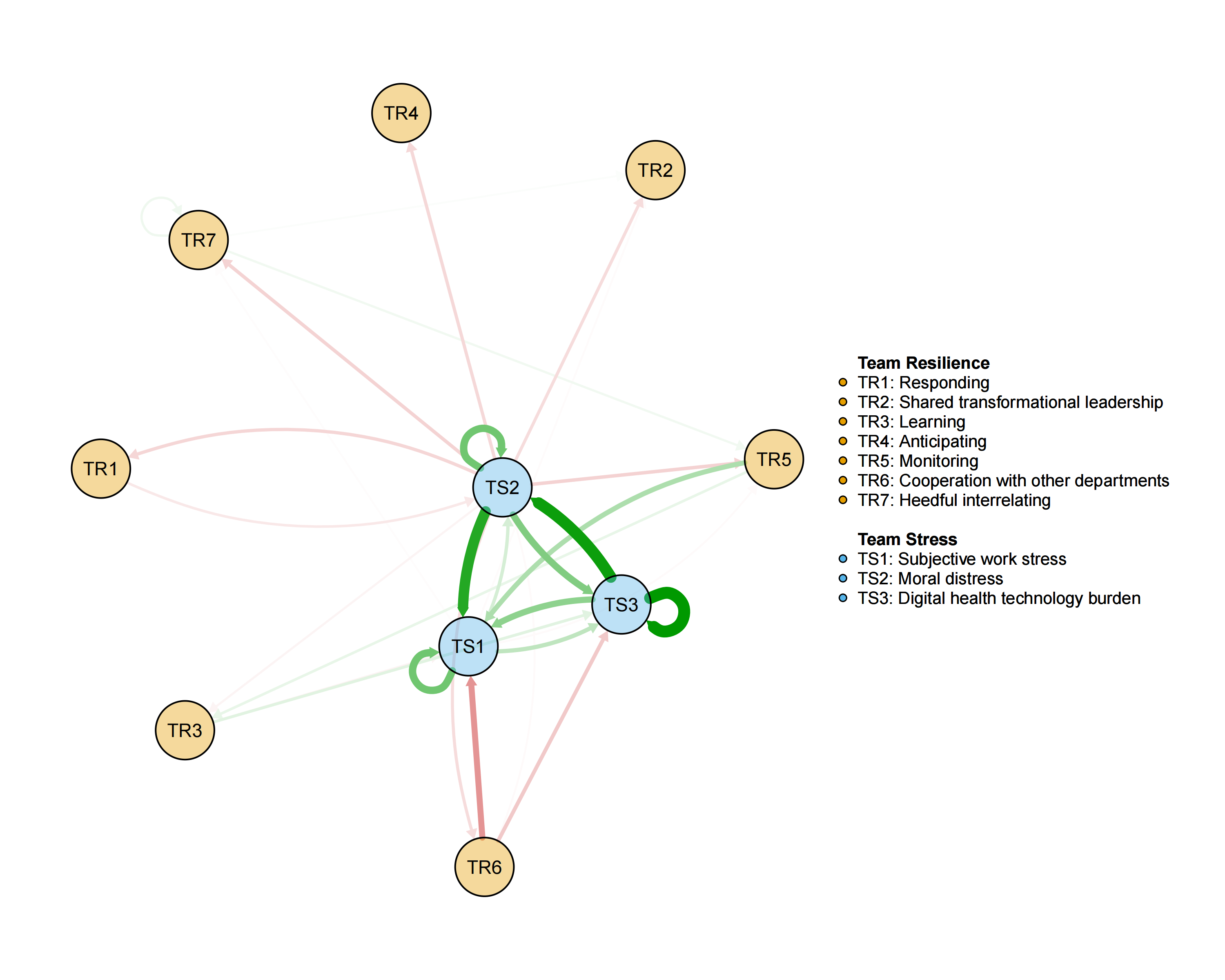


**Figure S1** | Structure of the estimated cross-lagged panel network (including all autoregressive and cross-lagged edges). Nodes represent dimensions of team resilience (TR, yellow) and team stress (TS, blue). Arrows indicate cross-lagged effects (prediction from T1 to T2), and self-loops indicate autoregressive effects. Green edges represent positive relationships; red edges represent negative relationships. Edge thickness represents the strength of edge weights.


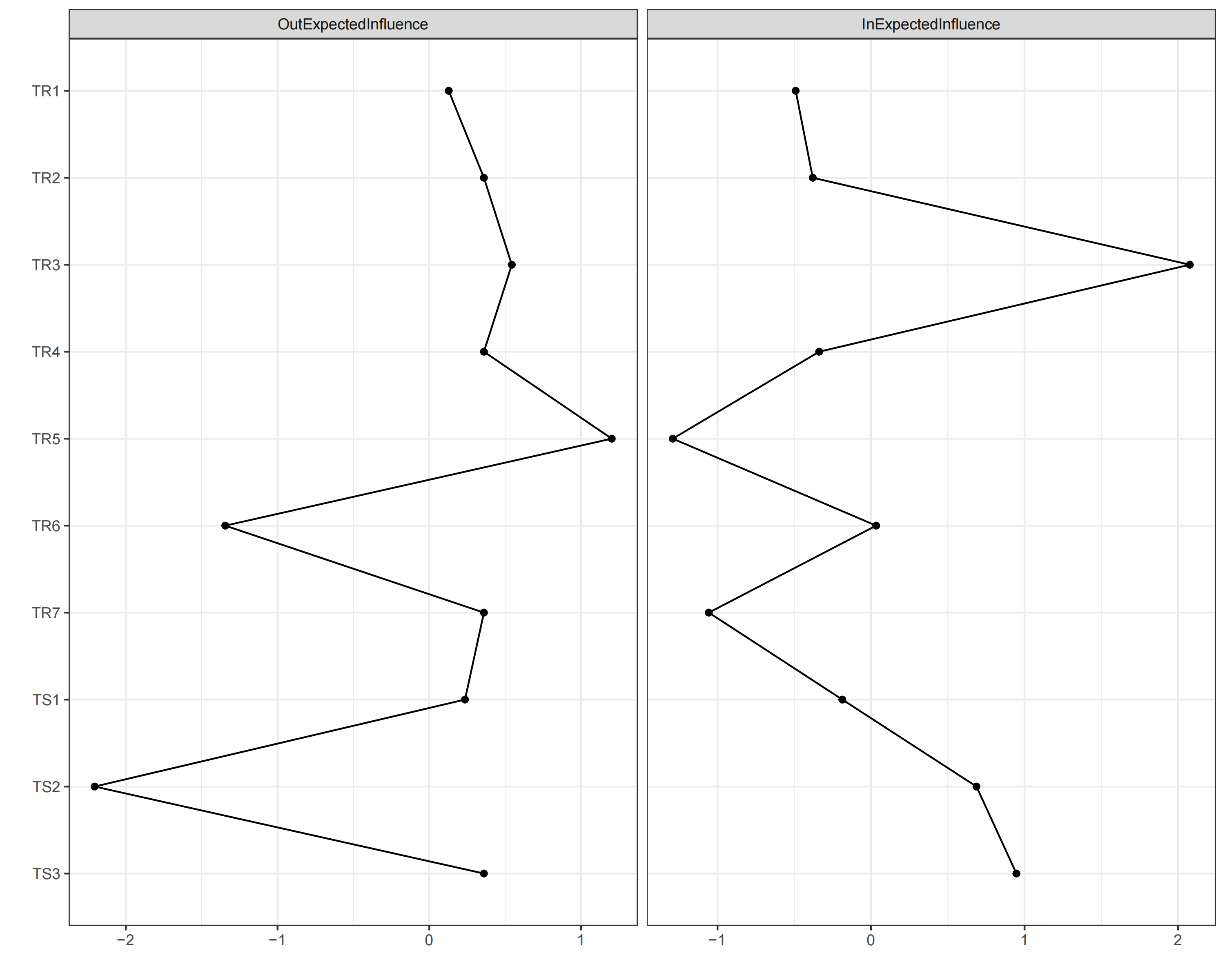


**Figure S2** | Standardized bridge out-expected influence and in-expected influence of the nodes. The figure displays the standardized indices for bridge out-expected influence (left) and bridge in-expected influence (right). Bridge out-expected influence quantifies the relative importance of a node at Time 1 in predicting the change of nodes in the other community at Time 2. Bridge in-expected influence quantifies the extent to which a node at Time 2 is predicted by the nodes in the other community at Time 1. Values are shown as z-scores. TR1-TR7: dimensions of Team Resilience; TS1-TS3: dimensions of Team Stress.


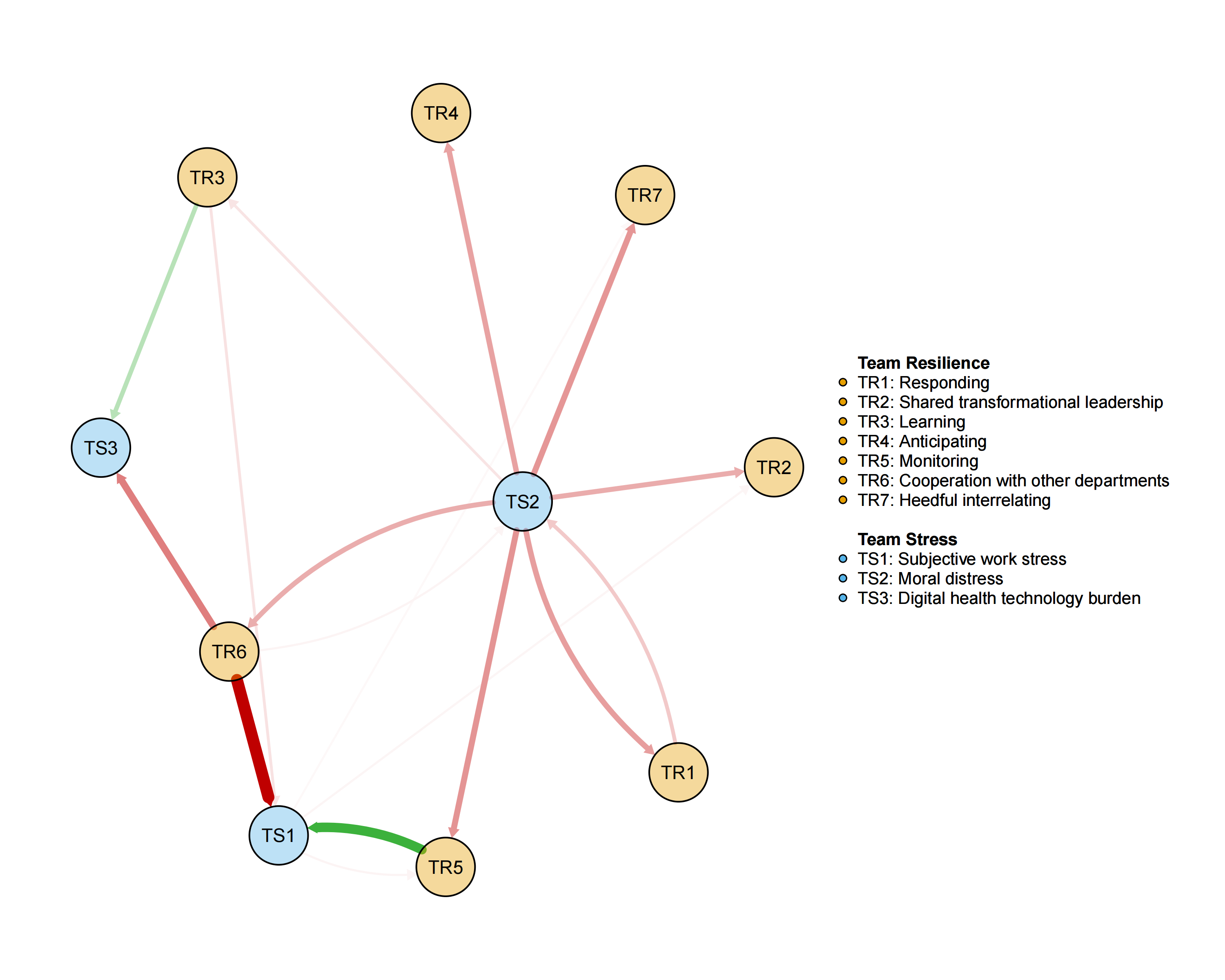


**Figure S3** | Structure of the estimated cross-lagged panel network (excluding cross-lagged edges in the same community and autoregressive edges). Nodes represent dimensions of team resilience (TR, yellow) and team stress (TS, blue). Arrows indicate cross-lagged effects (prediction from T1 to T2). Green edges represent positive relationships; red edges represent negative relationships. Edge thickness represents the strength of edge weights.


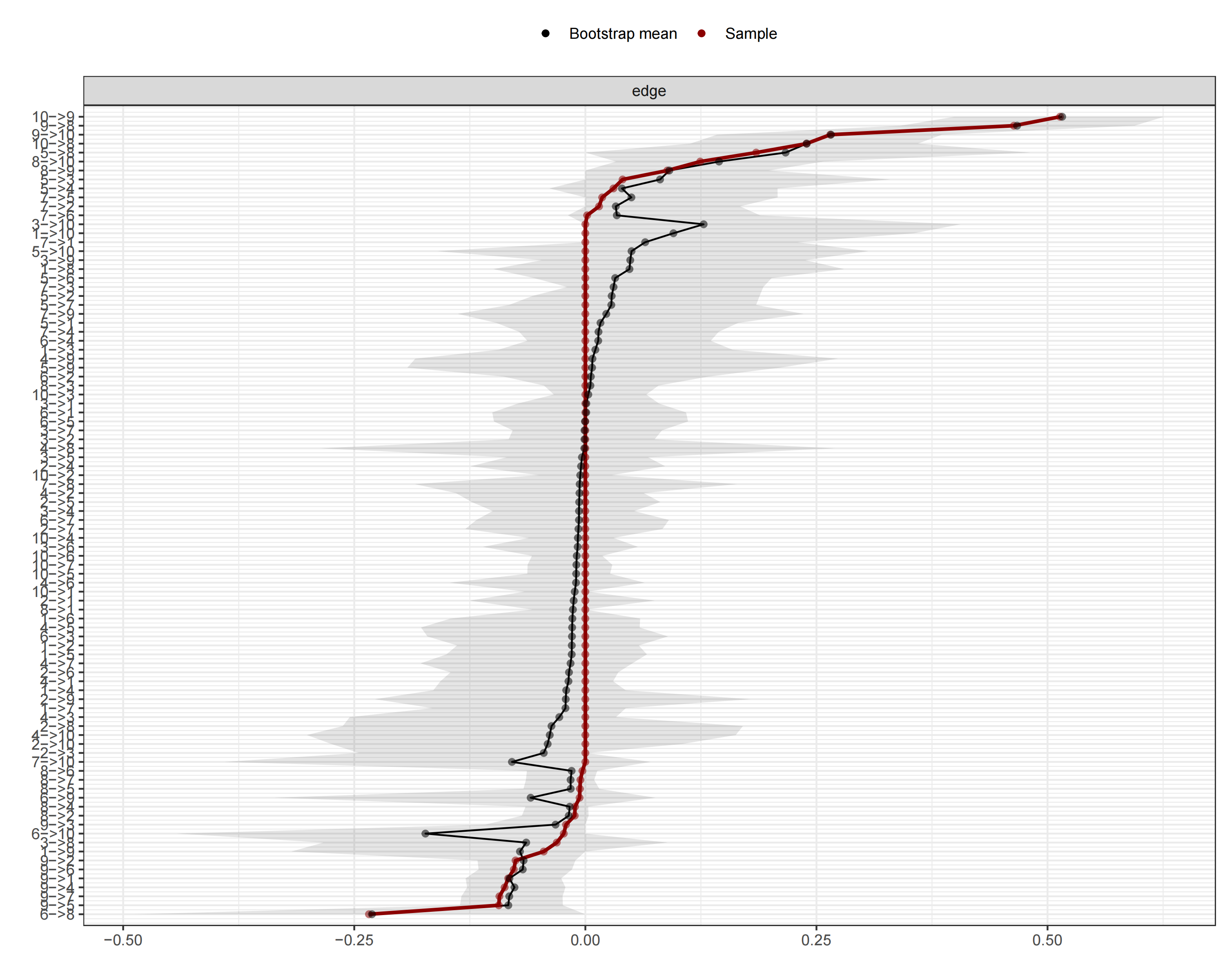


**Figure S4** | Accuracy of edge weight estimates for the cross-lagged network. The plot shows the 95% confidence intervals (gray area) of the edge weights based on 1,000 non-parametric bootstrap samples. The red line indicates the original sample values, and the black line represents the bootstrap mean. The y-axis displays the edges between nodes at Time 1 and Time 2 (where numbers 1-7 correspond to TR1-TR7, and 8-10 correspond to TS1-TS3).


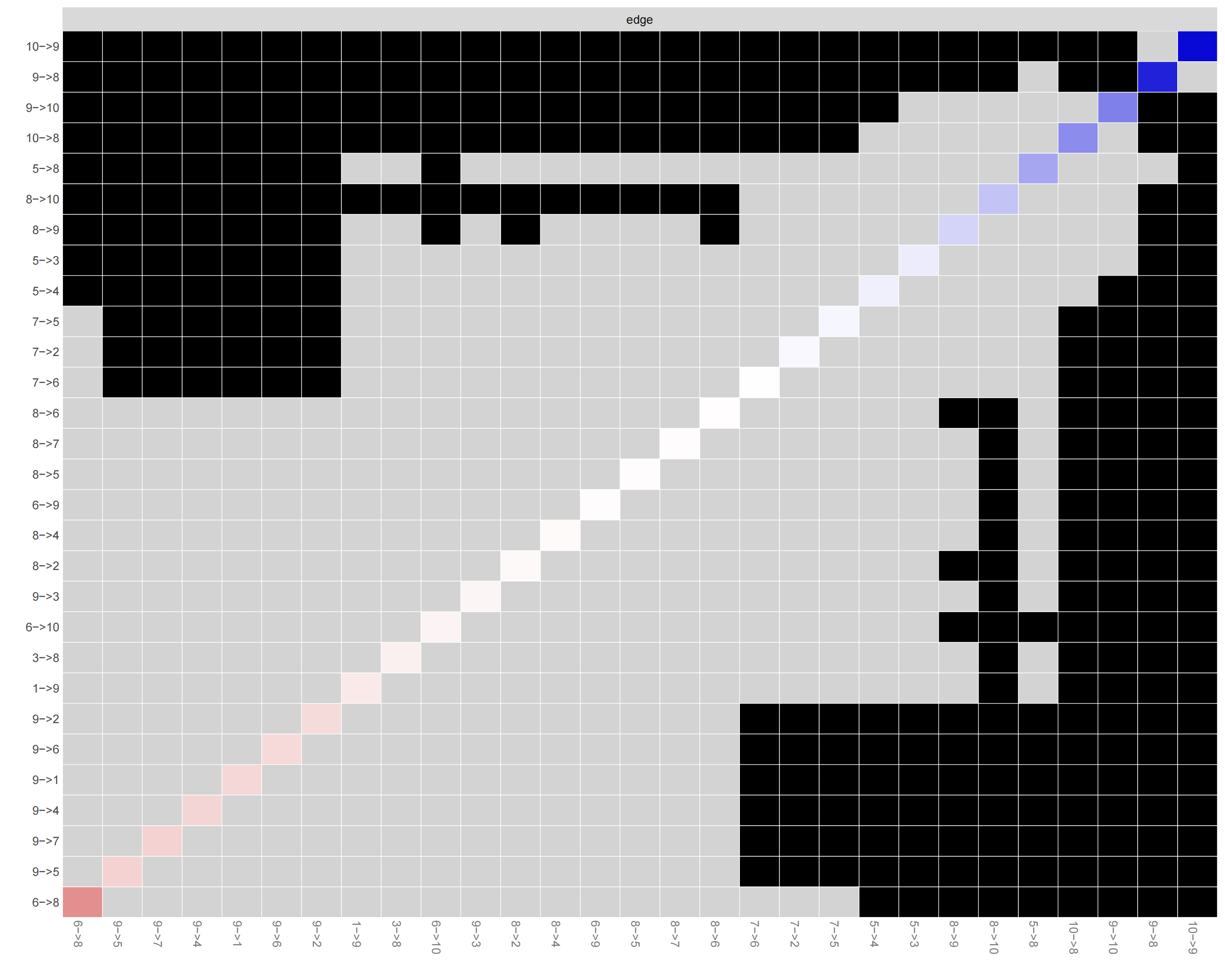


**Figure S5** | Bootstrapped difference test for edge weights in the cross-lagged network. This figure displays the results of the bootstrapped difference test (α = 0.05) for edge weights. Gray boxes indicate that the two corresponding edge weights do not differ significantly. Black boxes indicate that the two edge weights are significantly different from each other. The colored boxes on the diagonal represent the magnitude of each individual edge weight. Numbers 1-7 correspond to TR1-TR7, and 8-10 correspond to TS1-TS3.


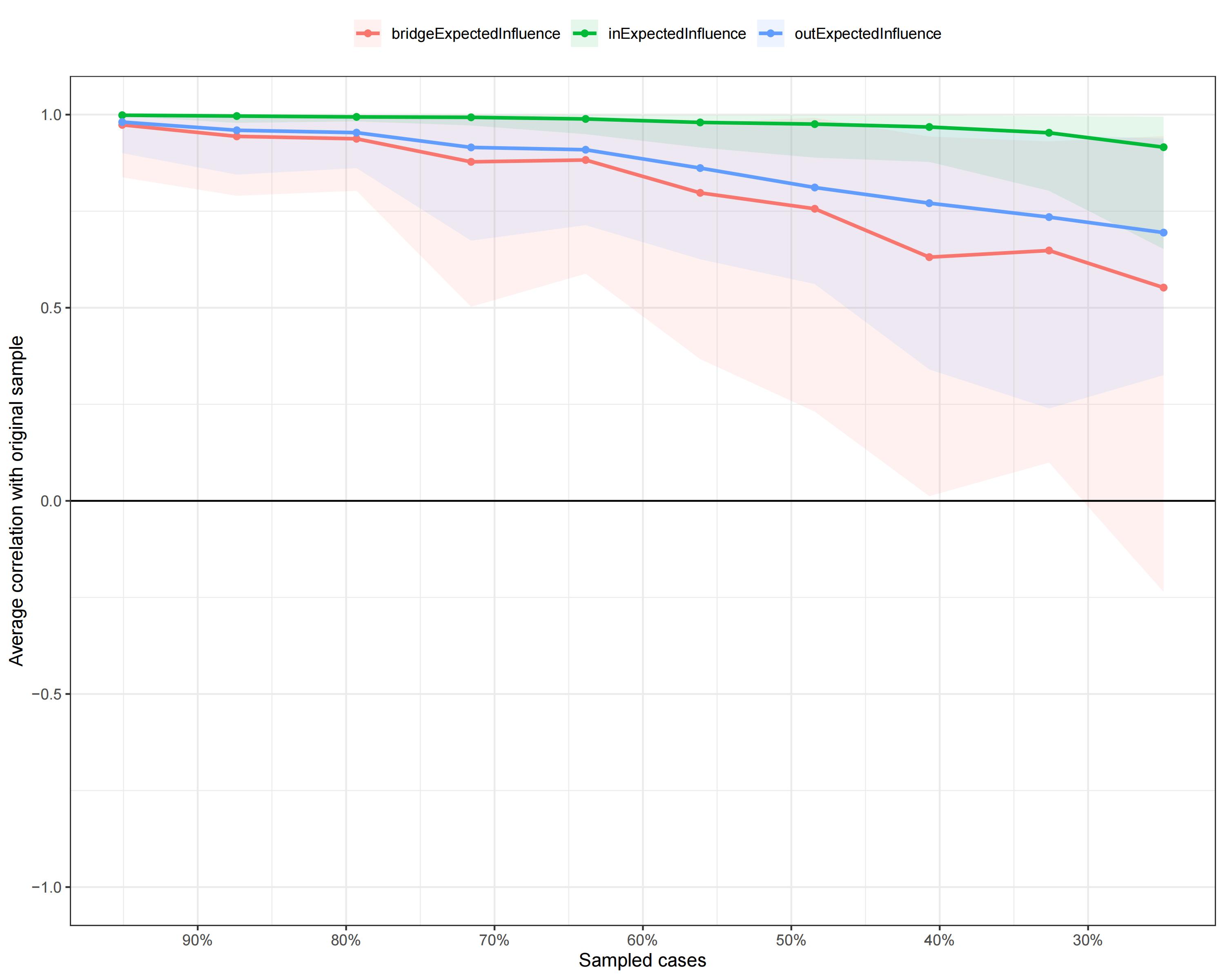


**Figure S6** | Stability of node expected influence using Case-dropping Bootstrap procedure. The figure illustrates the stability of three node influence indices: in-expected influence (green), out-expected influence (blue), and bridge-expected influence (red). The x-axis represents the percentage of cases dropped from the original sample, and the y-axis represents the average correlation between the centrality of the original network and the networks re-estimated with fewer cases. The shaded areas represent the 95% confidence intervals. The CS-coefficients (r = 0.7) for in-expected influence, out-expected influence, and bridge-expected influence were 0.751, 0.361, and 0.207, respectively.


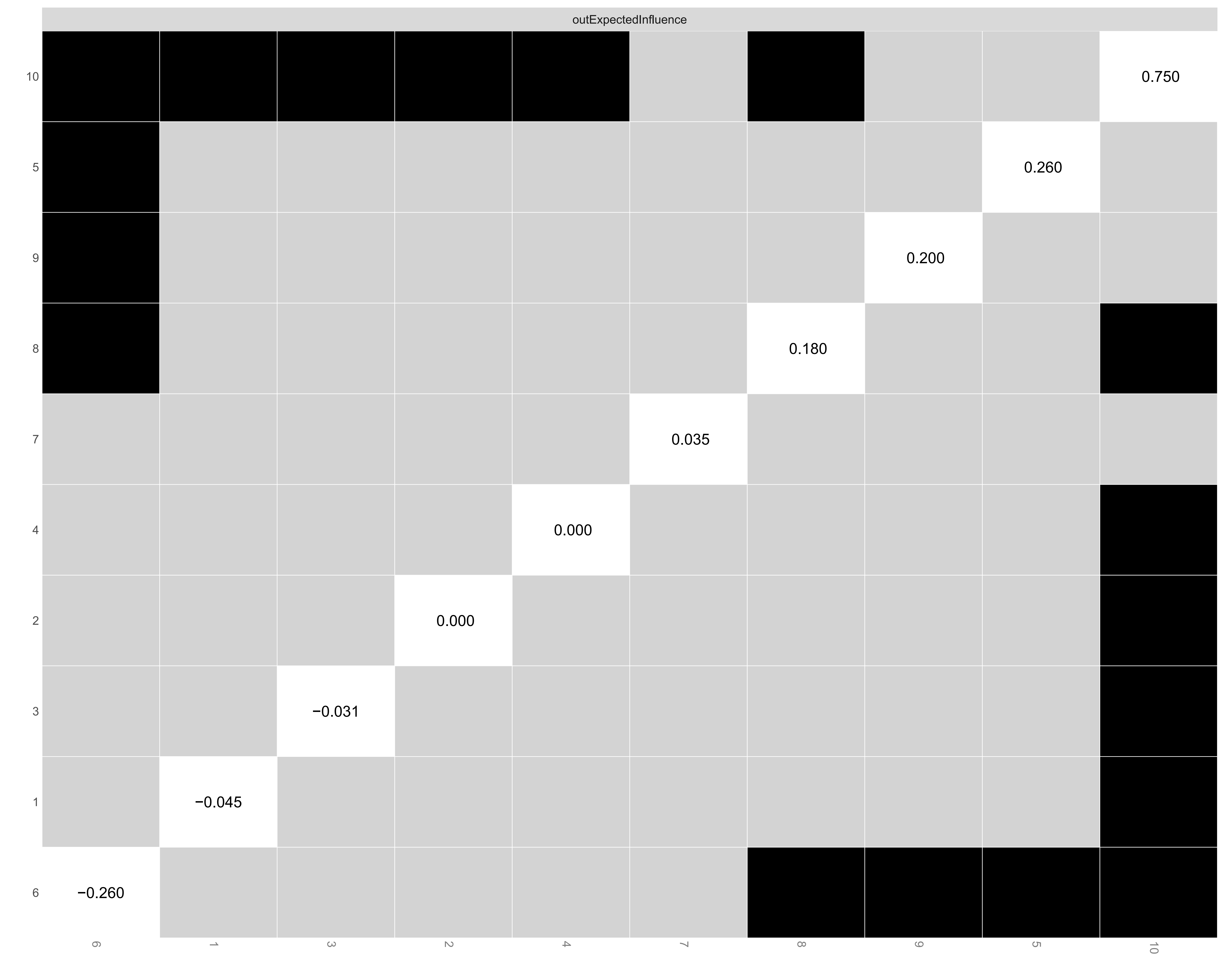

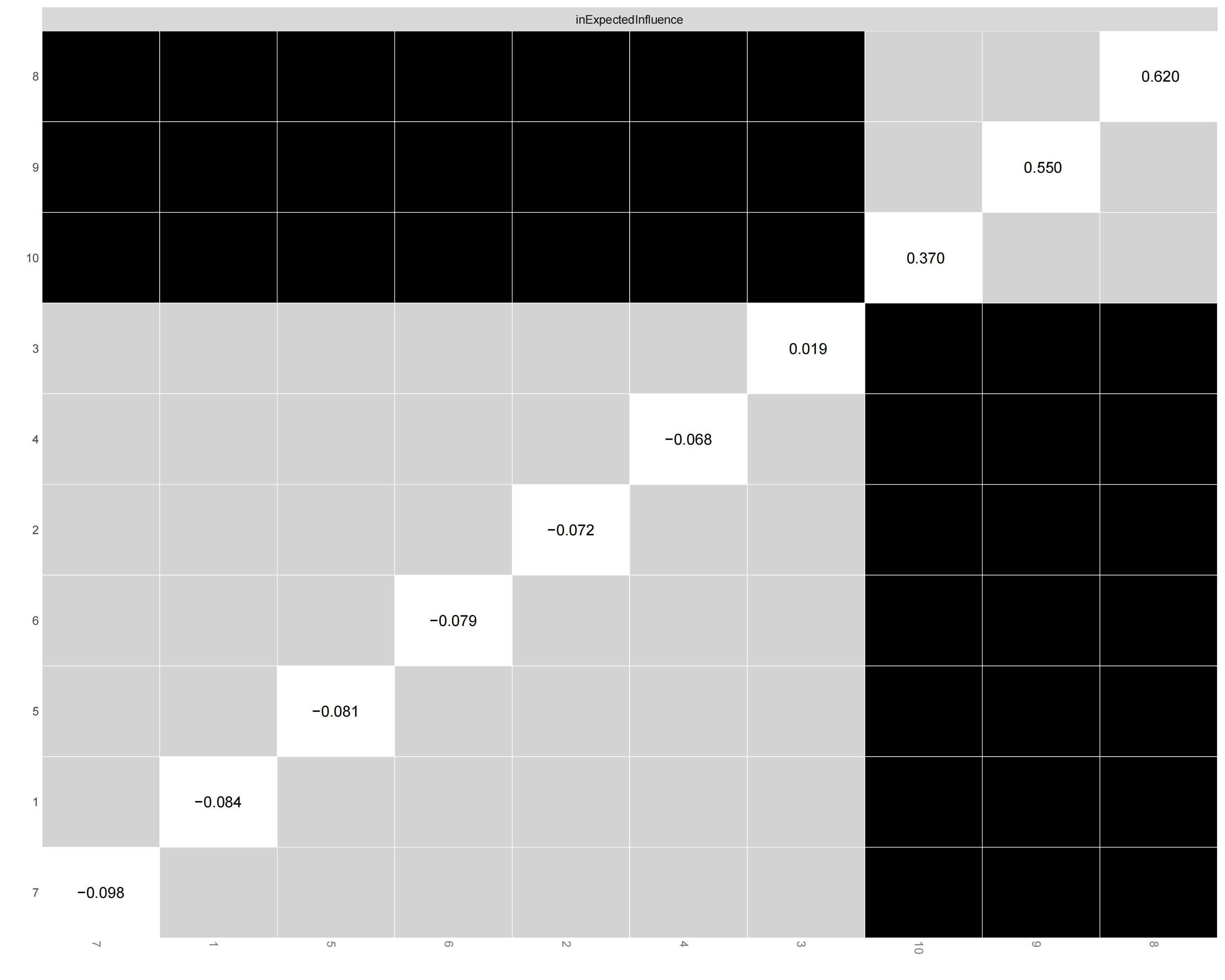

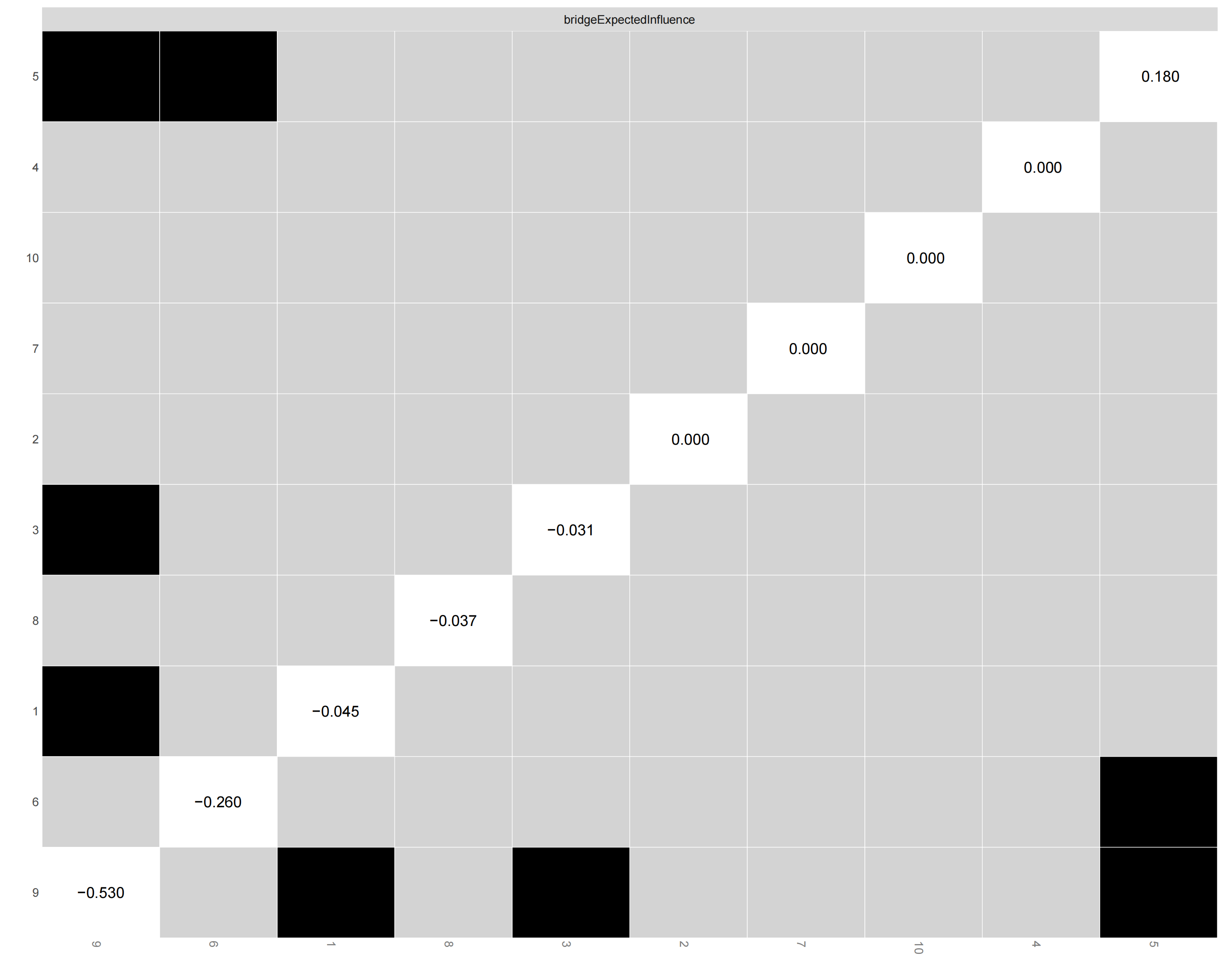


**Figures S7-S9** | Bootstrapped difference tests for node influence indices. These figures display the results of the bootstrapped difference tests (α = 0.05) for three node influence indices: Figure S7 for out-expected influence, Figure S8 for in-expected influence, and Figure S9 for bridge-expected influence. Gray boxes indicate that the two corresponding nodes do not differ significantly in their influence levels. Black boxes indicate a significant difference between the nodes. The white boxes on the diagonal show the raw value of the expected influence for each node (e.g., 0.750 for node 10 in S7). Numbers 1-7 correspond to TR1-TR7, and 8-10 correspond to TS1-TS3.
